# Taxonomy and control of *Trichoderma hymenopellicola* sp. nov. responsible for the first green mold disease on *Hymenopellis raphanipes*

**DOI:** 10.3389/fmicb.2022.991987

**Published:** 2022-09-29

**Authors:** Xiang-Yu Zeng, Xiao-Xiao Yuan, Ke-Qin Peng, Yin-Tao Pan, Ting-Jun Tan, Nan Wu, Feng-Hua Tian

**Affiliations:** ^1^Department of Plant Pathology, College of Agriculture, Guizhou University, Guiyang, China; ^2^Institute of Edible Fungi, Guizhou University, Guiyang, China; ^3^College of Life Sciences and Technology, Heilongjiang Bayi Agricultural University, Daqing, China

**Keywords:** fungicide, Hypocreaceae, novel taxa, pathogen, phylogeny, taxonomy

## Abstract

*Trichoderma* spp. are a group of widespread fungi with important applications in many aspects of human life, but they are also pathogens that cause green mold disease on mushrooms. During a survey of mushroom cultivation in Guizhou, China, five strains of *Trichoderma* from three different localities were isolated from soil in mushroom bags of *Hymenopellis raphanipes*. The typical morphology of having gregarious, reddish stromata and gregarious phialides and the results of phylogenetic analyses based on a combined dataset of RPB2, TEF, and ITS gene sequences demonstrated that these green-spored *Trichoderma* belong to a new taxon, *Trichoderma hymenopellicola*. Pathogenicity tests by covering fungal mycelial blocks or soil mixed with spore suspension in mushroom bags showed similar symptoms to those in the field, and the same fungal pathogen had been observed and re-isolated from these symptoms, which fulfill Koch’s postulates. A primary screening test of nine common fungicides indicated that prochloraz-manganese chloride complex and propiconazole are the top two effective fungicides inhibiting the pathogen, whereas the former was further indicated as a suitable fungicide to control *Trichoderma hymenopellicola*, with a high inhibition ratio to the pathogen and low toxicity to the mushroom.

## Introduction

*Hymenopellis raphanipes* (≡ *Oudemansiella raphanipes*) is a widely cultivated mushroom with edible and medicinal properties. With the increasing scale of cultivation, the annual yield of *Hymenopellis raphanipes* in China has exceeded 20,000 tons per year, with a corresponding output value of over 350 billion CNY. Meanwhile, various diseases began to appear during the cultivation, whereas researchers have paid more attention on optimizing its growth condition. It has been reported that cobweb disease caused by species of *Cladobotryum* on *Hymenopellis raphanipes* is a major problem during mushroom cultivation and can cause great yield losses (Liu et al., 2020; Qin et al., 2021). During our investigation of mushroom cultivation, the green mold disease was found on the soil surface and fruiting bodies of *Hymenopellis raphanipes* bags from three different localities. This disease has a 30–50% incidence and can cause mushroom bag rot and a decline in yield and quality.

Green mold disease is a common disease that often occurs during mushroom cultivation, with symptom of having green, villiform mycelium on the surface. *Trichoderma* spp. are the causal agent of green mold disease, which comprises a group of mostly saprobic fungi that are widespread in soil, healthy plants, wood, or other fungi. They are widely used to control fungal pathogens (Hasan et al., 2012; Liu et al., 2012; Abo-Elyousr et al., 2014; Poveda et al., 2019), produce antibiotics, enzymes, and biofuel (Degenkolb et al., 2008; Jun et al., 2011), and bioremediation xenobiotic compounds in water and soil (Katayama and Matsumura, 1993; Harman et al., 2004; Ezzi and Lynch, 2005). On the contrary, some species can cause economic losses in mushroom growing (Samuels et al., 2002; Kim et al., 2012) or infect humans (Kredics et al., 2003; Kuhls et al., 2010). To avoid the negative effects of *Trichoderma* on humans, fungicides like carbendazim, hexaconazole, metrafenone, prochloraz, tebuconazole, and thiophanate-methyl, can be used to prevent the occurrence and spreading of *Trichoderma* spp. (Madhavi et al., 2011; Kosanoviæ et al., 2015; Lukoviæ et al., 2021).

*Trichoderma* includes two groups of species with different colors of ascospores, i.e., hyaline and green ascospores. Green-spored *Trichoderma* species were first intensively studied by Chaverri and Samuels (2004). Afterward, Jaklitsch and Voglmayr (2015) divided them into six subclades, *viz*. Ceramicum, Chlorosporum, Harzianum, Helium, Spinulosum, and Strictipile, but this treatment has not been fully accepted by other researchers (Chen and Zhuang, 2017). Recently, Bustamante et al. (2021) conducted multilocus phylogenetic analyses and four DNA-based methods to delimit the *Trichoderma* species of the Harzianum lineage that comprises most green-spored species.

Morphologically, the new pathogen is characterized by green ascospores and colonies with reddish or yellowish stromata. Phylogenetically, it forms a distinct clade sister to *Trichoderma epimyces*, *T. priscilae*, *T. purpureum*, *T. rufobrunneum*, and *T. tenue*. To find a way of controlling the disease, the compatibility of the new pathogen with nine fungicide candidates was tested, and the top two most sensitive fungicides were further applied to strains from all three localities, as well as the mushroom strain. A fungicide that can inhibit the growth of the pathogen and is less toxic to the mushroom would be a suitable agent to prevent the occurrence and spreading of the disease.

## Materials and methods

### Fungal isolation

Mushroom bags with diseased *Hymenopellis raphanipes* were collected from mushroom cultivation bases at Jianhe (JH), Shuicheng (SC), and Zhenfeng (ZF) counties in Guizhou, China, during 2019 to 2021. Pathogens were isolated using the spread plate and tissue isolation method resulting in a total of five fungal isolates. Purified cultures were incubated on cornmeal dextrose agar (CMD), potato dextrose agar (PDA), and synthetic low nutrient agar (SNA) plates at 25, 30, and 35°C. Ex-type strains were deposited at the Culture Collection, Department of Plant Pathology, Guizhou University (GUCC). MycoBank number was registered for the new taxon (Crous et al., 2004).

### Morphological studies

Photographs of fresh stromata were taken using an ultra-depth of field stereomicroscope (digital microscope system Keyence VHX-7000) to illustrate the macrostructures. Sections were made using a stereomicroscope (Leica S9i) and mounted in water or a rehydrated 5% KOH solution. Photomicrographs of perithecia, asci, ascospores, conidiophores, conidia, and phialides were taken with a compound light microscope (Zeiss Scope 5 with color camera AxioCam 208) to observe the morphological characteristics. All measurements of the observed structures were made with ZEN2 (blue edition) software.

### Pathogenicity assays

A pathogenicity test was conducted by inoculating fungal mycelial blocks and spore suspensions of fungal strains isolated from JH, SC, and ZF on the soil surface of 90-day-old mushroom bags and fruiting bodies of *Hymenopellis raphanipes* all groups were further incubated at room temperature. PDA blocks and distilled water were used in the control check (CK) to replace the mycelial blocks and spore suspensions. Photographs of bags were taken after 1, 7, and 14 days, respectively, to check if any green mycelium occurred. Then, the fungal pathogen was re-examined and re-isolated from the diseased area to fulfill Koch’s postulates.

An antagonistic experiment was also conducted by inoculating mycelial blocks of the pathogen and mushroom on PDA plates and incubating at room temperature. When the colony of pathogen and mushroom overlapped, photographs of the petri dishes and photomicrographs of the overlapping hyphae were taken to see if there was any interaction between the two species.

### DNA extraction and sequencing

Total genomic fungal DNA was extracted by a CwBiotech Plant Genomic DNA Kit (Changping, Beijing, China) following the manufacturer’s protocol. The internal transcribed spacer (ITS) along with the 5.8S ribosomal rDNA, partial translation elongation factor 1-α (TEF), and RNA polymerase II second largest subunit (RPB2) were amplified with the primer pairs ITS5/ITS4, EF1-728F/TEF1LLErev, and fRPB2-5F/fRPB2-7cR, respectively (White et al., 1990; Carbone and Kohn, 1999; Liu et al., 1999; Jaklitsch et al., 2005).

Polymerase chain reaction (PCR) reactions were employed in a 25 μl reaction mixture containing 1.6 μl dNTP mix (2.5 mM/μl), 0.2 μl of Taq polymerase (5 U/μl), 2 μL polymerase buffer (10 × /μl), 1 μl forward and reward primers (10 μM/μl), and 1 μl DNA template. Amplifications were performed in a T100™ Thermal Cycler (BIO-RAD), which was programmed for an initial denaturation at 95°C for 3 min followed by 34 cycles of 1 min at 95°C, 30 s at 55°C and extension at 72°C for 1 min, and a final extension at 72°C for 10 min. PCR products were sequenced by using the same PCR primers used in amplification reactions by Sangon Biotech (Shanghai) Co., Ltd.

### Phylogenetic analyses

Sequences of each gene generated from forward and reverse primers were assembled with BioEdit v.7.2.5 (Hall, 1999), and consensus sequences were then combined with related sequences downloaded from GenBank (Supplementary Table 1). Each gene dataset was aligned separately by Mafft v7.187 (Katoh and Standley, 2013), and manually aligned where necessary. The nucleotide substitution model for each gene was determined by the Bayesian information criterion (BIC) using jModelTest v2.1.6 (Darriba et al., 2012). Phylogenetic trees based on a concatenated dataset of RPB2, TEF, and ITS, generated by SequenceMatrix v1.7.8 (Vaidya et al., 2011), were constructed using maximum likelihood (ML) and Bayesian inference (BI) analyses at the CIPRES Web Portal (Miller et al., 2010). ML was performed using the “RAxML-HPC BlackBox” tool (Stamatakis, 2014). The Markov Chain Monte Carlo (MCMC) algorithm for BI with two parallel runs of four chains was performed using the “MrBayes on XSEDE” tool (Ronquist et al., 2012). Trees were sampled every 100 generations, and runs were stopped automatically when the average standard deviation of split frequencies fell below 0.01. A 50% majority rule consensus tree was summarized after discarding the first 25% of samples. The resulting trees were visualized in FigTree v1.4.3 (Rambaut, 2016).

### Fungicide sensitivity assays

The type strain of the new collection was first used to conduct primary fungicide sensitivity tests *in vitro* against four biological fungicides and five chemical fungicides with five different gradient concentrations based on the instructions and our preliminary tests (Table 1). Each fungicide was applied to PDA plates with three replicates, and the same amount of distillation water was applied in CK. Mycelial blocks of 5 mm in diameter from 6-day-old cultures were placed in the center of the plates and incubated at 25°C. Afterward, the diameters of colony (D) were measured after 6-day incubation, and the inhibition ratio (IR = 1–D/D_CK_) was calculated. Linear regression analysis and the half maximal effective concentration (EC_50_) were calculated using DPS V18.10, and the significance of the difference was calculated by Duncan’s new multiple range test.

**TABLE 1:** Fungicide candidates and their concentration used for primary fungicide sensitivity assay.

Fungicide	Group 1 (mg/L)	Group 2 (mg/L)	Group 3 (mg/L)	Group 4 (mg/L)	Group 5 (mg/L)
Berberine	125	25	5	1	0.2
Carvacrol	500	100	20	4	0.8
Eugenol	30	6	1.2	0.24	0.048
Metalaxyl	30	6	1.2	0.24	0.048
Osthole	50	10	2	0.4	0.08
Trifloxystrobin + Tebuconazole	150	30	6	1.2	0.24
Phenazine-1-carboxylic acid	100	20	4	0.8	0.16
Prochloraz-manganese chloride complex	0.2	0.04	0.008	0.0016	0.00032
Propiconazole	4	1	0.25	0.0625	0.01563
Thiophanate-methyl	500	100	20	4	0.8

After the primary test, representative strains from the other two collection sites were replicated three times, as well as the mushroom strain, to further test their sensitivity to the top two most effective fungicides.

## Results

### Pathogenicity tests

**FIGURE 1:**
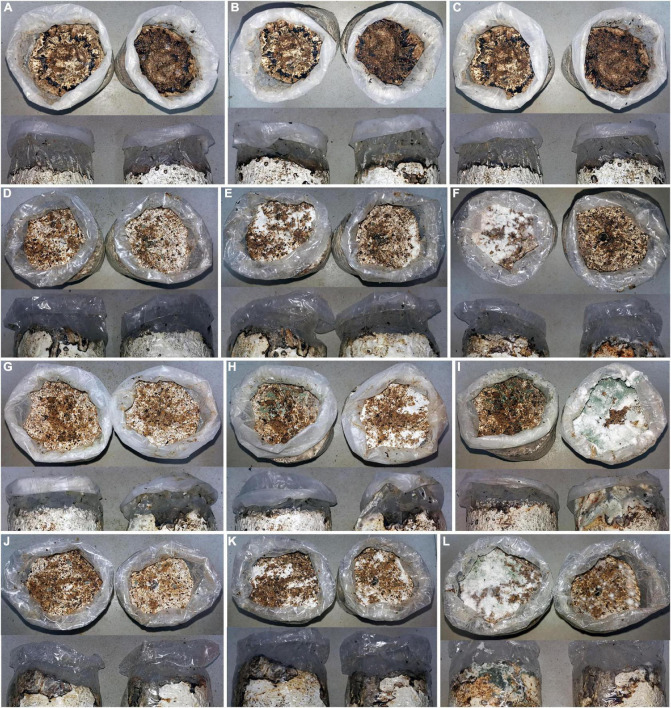
Pathogenicity test of *Trichoderma hymenopellicola* by covering its mycelial blocks (upper) and soil mixed with spore suspension (lower). **(A–C)** CK group after 1, 7, and 14 days. **(D–F)** JH group after 1, 7, and 14 days. **(G–I)** SC group after 1, 7, and 14 days. **(J–L)** ZF group after 1, 7, and 14 days.

Both soil inoculating groups of covering mycelial blocks and soil mixed with spore suspension of *Trichoderma hymenopellicola* exhibited similar symptoms of green mold disease in the field after 7 days (Figures 1D–L), while the control group do not have (Figures 1A–C). The white mycelium can be observed on the surface of mushroom bags after 3–5 days and spread fast, which covered the whole surface of substrate and turned green within 10 days. The rate of *Trichoderma hymenopellicola* infecting mushroom bags is about 50%, which is similar to its incidence in the field. The same fungal pathogen had been observed and re-isolated from these symptoms, which fulfils Koch’s postulates.

**FIGURE 2:**
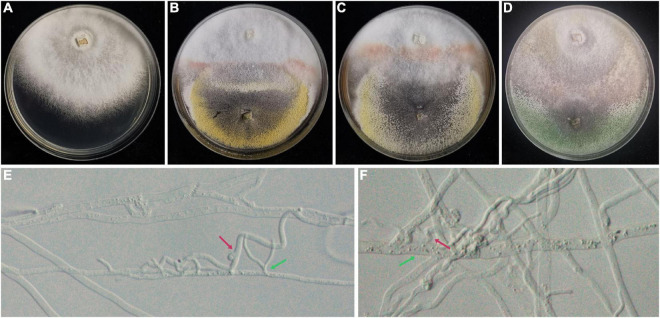
Antagonistic experiment between strains of *Trichoderma hymenopellicola* and *Hymenopellis raphanipes* on PDA. **(A)** Mushroom colony (CK). **(B)** Mushroom colony with pathogen from JH. **(C)** Mushroom colony with pathogen from SC. **(D)** Mushroom colony with pathogen from ZF. **(E,F)** Hyphae of *Trichoderma hymenopellicola* (green arrow) and abnormal mushroom hyphae (red arrow).

The result of an antagonistic experiment demonstrated that hyphae of *Trichoderma hymenopellicola* can cause hyphae of *Hymenopellis raphanipes* to grow abnormally (Figures 2E,F), and the overlapping part of two colonies does not form a clear boundary (Figures 2A–D). On the contrary, infections were not observed in the fruiting body inoculating group.

### Phylogenetic analyses

**FIGURE 3:**
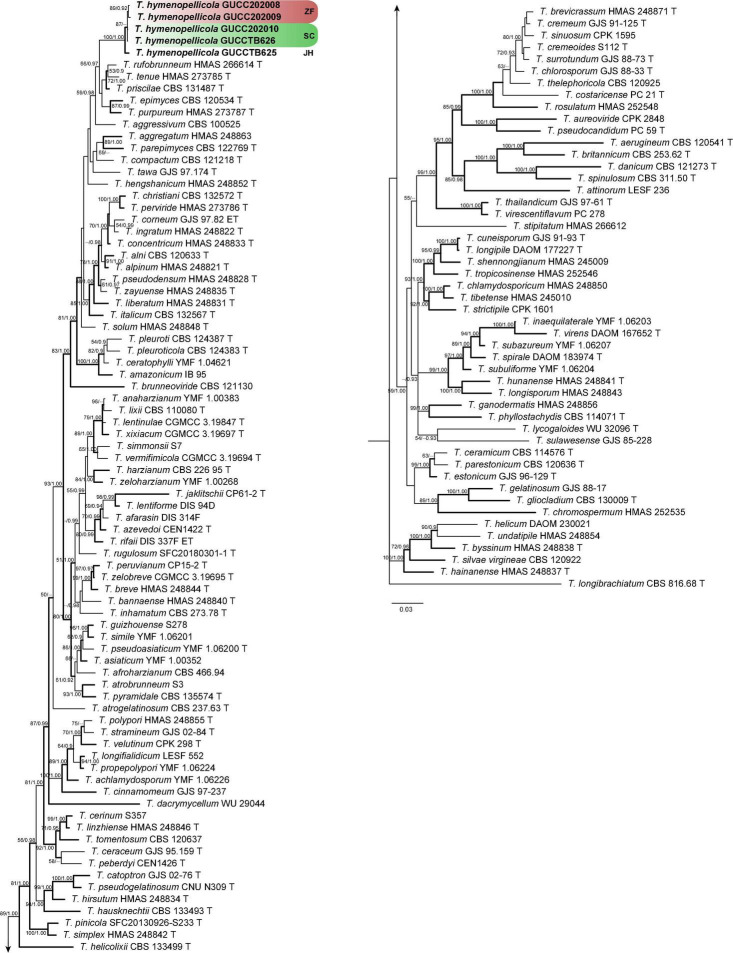
Phylogram generated from Bayesian inference based on combined ITS, RPB2, and TEF sequence data of green-spored *Trichoderma* species with *T. longibrachiatum* as the outgroup. Maximum likelihood bootstrap support (BS) above 50% and Bayesian posterior probabilities (PP) above 0.9 are shown at nodes. Clades with strong support (BS ≥ 70, PP ≥ 0.95) are indicate in bold. New sequences obtained from this study are in bold. ‘T’ represents ex-type strains.

The concatenated dataset (Supplementary Table 1) consists of 129 strains and 2,922 unambiguously aligned sites (ITS, 609; RPB2, 1024; and TEF, 1289). The best-fit substitution model of each gene is TPM1uf + I + G (RPB2 and TEF) and ITS (TPM2uf + I + G). The RAxML analysis of the combined dataset yielded a best-scoring tree with a final ML optimization likelihood value of –35239.670452. Estimated base frequencies are as follows: *A* = 0.233134, *C* = 0.285526, *G* = 0.253003, and *T* = 0.228336; substitution rates AC = 1.134637, AG = 4.477934, AT = 1.149518, CG = 1.048786, CT = 6.335323, and GT = 1.000000; proportion of invariable sites *I* = 0.544721; and gamma distribution shape parameter α = 0.951765. The Bayesian analysis ran 29,64,000 generations before the average standard deviation for split frequencies reached 0.00998. The analysis generated 59,282 trees in total, from which 44,462 were sampled after burn-in, and the 99% credible set contains 35,309 trees. Our new strains belong to a distinct clade that is genetically distant from *Trichoderma epimyces*, *T. priscilae*, *T. purpureum*, *T. rufobrunneum*, and *T. tenue*, and is divided into three subclades represented by strains from the three localities (Figure 3).

### Fungal taxonomy

**FIGURE 4:**
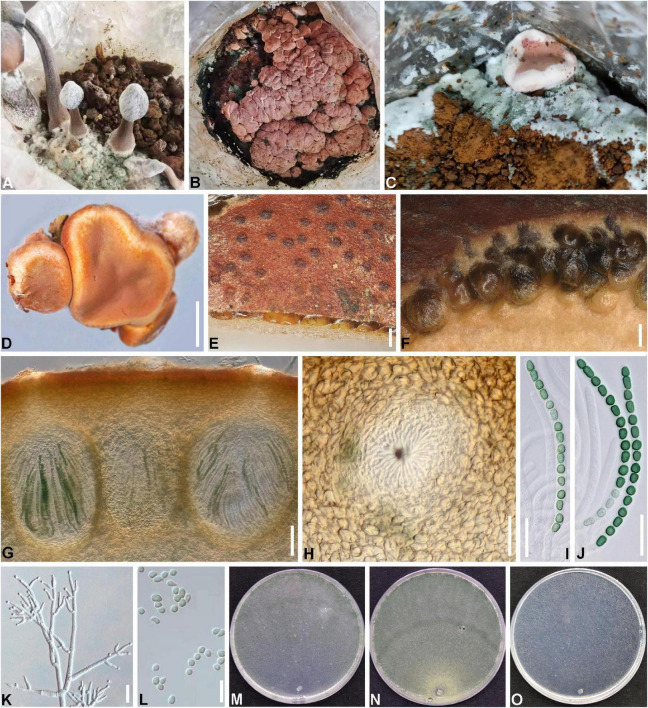
*Trichoderma hymenopellicola* (HGUP 202007, GUCC 202010). **(A–C)** Disease in the field. **(D)** Fresh stromata. **(E)** Ostiolar dots on stromata surface. **(F)** Cortical and subcortical tissue. **(G)** Cortical and subcortical tissue in section. **(H)** Ostiole. **(I,J)** Asci with ascospores. **(K)** Conidiophores and phialides. **(L)** Conidia. **(M)** Cultures on CMD (5 days). **(N)** Cultures on PDA (5 days). **(O)** Cultures on SNA (4 days). Scale bars: **(D)** 1,000 μm, **(E,F)** 100 μm, **(G)** 50 μm, **(H–K)** 20 μm, **(L)** 10 μm.

*Trichoderma hymenopellicola* X.Y. Zeng, X.X. Yuan and F.H. Tian, sp. nov. Figure 4.

MycoBank number: 840876.

Etymology: In reference to its occurrence in *Hymenopellis raphanipes* bags (Figures 4A–C).

*Stromata* 1–15 mm in diameter, 1–10 mm thick (*n* = 10), mostly gregarious, discoid or undulate, with yellowish margin and pale red, depressed center when young, becoming reddish with rugose surface when mature (Figure 4D). *Ostiolar dots* are umbilicate brown. *Rehydrated stromata* are larger than dry ones, the surface is smooth, and they become purple in 5% KOH (Figure 4E). *Cortical layer* comprising thick-walled, brown cells with *textura angularis* (Figure 4F). *Perithecia* 185–208 × 124–179 μm (*n* = 20), flask-shaped or subglobose, crowded (Figure 4G). *Peridium* 9–14 μm thick at sides, 13–22 μm thick at base (*n* = 30), light brown. *Ostioles* 87–102 × 33–44 μm (*n* = 30) (Figure 4H). *Asci* 87–98 × 5–6 μm (*n* = 30), including a 9–19 μm long stipe, 16-spored, cylindrical, hyaline (Figures 4I,J). *Ascospores* 5–7 × 3–5 μm (*n* = 50), 1-seriate, ellipsoid to globose, green, and verrucose.

#### Culture characteristics

Growth optimum at 25°C and no growth at 35°C on all media. On CMD after 72 h 49–50 mm, mycelium covering the plate after 4 days (Figure 4M). On PDA after 72 h 45–49 mm, mycelium covering the plate after 5 days (Figure 4N). *Colony* hyaline, greenish, yellowish, or pink when old, dense, circular, margin well defined and stellate due to parallel, aggregated surface hyphae. *Aerial hyphae* numerous, thin, and complexly branched, forming radial and circular strands. *Conidiophores* 1–3 level are branched and tapered at the tips (Figure 4K). *Phialides* are mostly gregarious, subfusiform, or cylindrical. *Conidia* are subglobose, smooth, hyaline, and scar indistinct, with no or few minute guttules (Figure 4L). On SNA, mycelium had covered the plate for 72 h (Figure 4O). *Colonies* are hyaline, thin, and circular. *Aerial hyphae* are scant and thin.

#### Material examined

China, Guizhou, Liupanshui, Shuicheng district, on soil surfaces of *Hymenopellis raphanipes* bags, March 2021, X-XY (**holotype** HGUP20071; **ex-type culture** GUCC202010; **culture** GUCCTB626). China, Guizhou, Southeast Guizhou Autonomous prefecture, Jianhe County, on the soil surface of *Hymenopellis raphanipes* bags, July 2021, X-XY (GUCCTB625). China, Guizhou, Southwest Guizhou Autonomous prefecture, Zhenfeng County, on the soil surface of *Hymenopellis raphanipes* bags, January 2020, X-XY (cultures GUCC202008 and GUCC202009).

#### Notes

Phylogenetically, our new collections cluster with *Trichoderma aggressivum*, *T. epimyces*, *T. priscilae*, *T. purpureum*, *T. rufobrunneum*, and *T. tenue* in the Harzianum lineage with high posterior probability (Figure 3), but with at least 2% (8/607 nucleotides, 5 gaps) difference in ITS, 4% (32/903 nucleotides, no gaps) difference in RPB2, and 5% (27/575 nucleotides, 5 gaps) difference in TEF. Morphologically, our new collections are most similar to *T. epimyces* in the size of stromata, perithecia, asci, and ascospores but have deeper color of stromata and ascospores, less pigment on media, and faster growth rate on media (Jaklitsch, 2009). The difference in our collections with *T. epimyces* is more than 2% (11/574 nucleotides, 3 gaps) in ITS, 4% (33/933 nucleotides, no gaps) in RPB2, and 6% (32/571 nucleotides, 6 gaps) in TEF.

### Fungicide sensitivity *in vitro*

The compatibility of *Trichoderma hymenopellicola* GUCC202010 to the nine fungicide candidates with regression equations is listed in Table 2, among which prochloraz-manganese chloride complex and propiconazole are the top two effective fungicides inhibiting the mycelium of *Trichoderma hymenopellicola* with EC_50_ less than 0.05 mg/L, while Osthole is found to be the best biological fungicide (Table 2).

**TABLE 2:** Results of fungicide sensitivity assay of strain GUCC202010.

Fungicide	Regression equation	EC_50_ (mg/L)	Correlation coefficient
Berberine	*Y* = 4.001 + 0.697X	27.066	0.982
Carvacrol	*Y* = 0.816 + 2.700X	35.446	0.986
Eugenol	*Y* = 4.531 + 0.435X	11.98	0.961
Osthole	*Y* = 4.017 + 0.993X	9.78	0.914
Phenazine-1-carboxylic acid	*Y* = 5.029 + 0.347X	0.824	0.976
Prochloraz-manganese chloride complex	*Y* = 6.672 + 0.644X	0.003	0.954
Propiconazole	*Y* = 8.844 + 2.707X	0.038	0.962
Thiophanate-methyl	*Y* = 3.429 + 1.051X	31.289	0.994
Trifloxystrobin + Tebuconazole	*Y* = 3.978 + 1.051X	9.387	0.994

The compatibility from high to low of strains from all three localities to the prochloraz-manganese chloride complex and propiconazole is SC, ZF, and JH, respectively (Table 3). The toxicity of these two chemicals to *Hymenopellis raphanipes* strains is also listed in Table 3. The results showed that the prochloraz-manganese chloride complex is a suitable chemical agent to control *Trichoderma hymenopellicola*, with a high inhibition ratio for the pathogen and low toxicity to the mushroom.

**TABLE 3:** Sensitivity of mushroom strain and *Trichoderma* strains to prochloraz-manganese chloride complex and propiconazole.

Strains	Regression equation	EC_50_ (mg/L)	Correlation coefficient
**Prochloraz-manganese chloride complex**			
Mushroom	Not sensitive		
GUCCTB625	*Y* = 6.1680 + 0.3570X	0.0005	0.9594
GUCC202009	*Y* = 10.3866 + 2.0727X	0.0025	0.9364
GUCC202010	*Y* = 6.6722 + 0.6437X	0.0025	0.9544
**Propiconazole**			
Mushroom	*Y* = 5.1979 + 0.7385X	0.5395	0.9772
GUCCTB625	*Y* = 7.6454 + 1.4557X	0.0152	0.9143
GUCC202009	*Y* = 7.6386 + 1.5262X	0.0187	0.9265
GUCC202010	*Y* = 8.8438 + 2.7073X	0.038	0.9624

## Discussion

*Hymenopellis raphanipes* was first described by Berkeley (1850) in India and is widely distributed in China (Hao et al., 2016). It had been previously misidentified as *H. furfuracea*, *H. radicata*, *Termitomyces fuliginosus*, or *T. badius*, until Hao et al. (2016) clarified its taxonomic placement based on both morphology and phylogeny. In recent years, cultivation scales of *Hymenopellis raphanipes* in China have increased rapidly, accompanying a series of diseases resulting in great yield losses, such as cobweb disease (Liu et al., 2020; Qin et al., 2021). Green mold disease is also a very common disease in mushroom cultivation, typical of superficial, green, and villiform mycelium. It can be either a competitive disease that antagonizes the normal growth of mushrooms or an infectious disease that causes the fruiting bodies or mycelium of mushrooms to grow abnormally, or both (Bian, 2013). In our study, only inoculations of soil showed the typical symptoms of green mold disease, while infections were not observed in the fruiting body inoculating experiment. However, the hyphae of the pathogen can affect the growth of mushroom mycelium, indicating that the infection is susceptible at the early stages of mushroom cultivation. In addition, the color of *Trichoderma hymenopellicola* colonies on PDA plates can also be yellow or pink, besides green in the field, which may be the result of environmental changes.

*Trichoderma hymenopellicola* is the first report that *Trichoderma* spp. cause green mold disease on *Hymenopellis raphanipes*. The new species has gregarious, larger, reddish stromata, gregarious phialides, and a fast-growing rate on media, with a preference of growing on SNA. The current classification system of *Trichoderma* relies on the phylogeny, as most species were isolated from environments, such as soil, and lack sexual morphs (Chen and Zhuang, 2017; Zheng et al., 2021). However, the recognition of subclades is empirical and does not have a compatible standard, and the TEF sequence data are not all from the same region. In this study, we included 128 *Trichoderma* species of the Harzianum lineage and green-spored species according to previous studies to better interpret their phylogenetic relationship (Zhu and Zhuang, 2015; Chen and Zhuang, 2017; Bustamante et al., 2021; Zheng et al., 2021). The topology of phylogenetic trees based on single gene and concatenated genes is similar, except that *Trichoderma aggressivum* does not cluster in our new collection in the tree generated from the RPB2 dataset. In the future, more studies with full morphological illustration and description are needed for further clarifying the interspecific relationship and fast identification of *Trichoderma* species.

Fungicides, such as carbendazim, hexaconazole, metrafenone, prochloraz, tebuconazole, and thiophanate-methyl, have been used to prevent the occurrence and spreading of *Trichoderma* spp., whereas prochloraz is the most effective one with an EC_50_ less than 0.4 mg/L (Madhavi et al., 2011; Kosanoviæ et al., 2015; Lukoviæ et al., 2021). In our study, we tested its manganese chloride complex, which is much more effective than prochloraz itself with an EC_50_ less than 0.005 mg/L and is non-toxic to the mushroom. As both an infectious and competitive disease, species of *Trichoderma* grow much faster than mushrooms, and infection at the early stage will cause no fruiting in mushroom bags. As a result, control of the green-mold disease should be done as early as possible during cultivation.

In this study, we reported the first green mold disease on *Hymenopellis raphanipes*. Its causal agent was further confirmed as a new species, *Trichoderma hymenopellicola* sp. nov., based on morphology and phylogeny. The new pathogen can infect the mycelium of *Hymenopellis raphanipes* at an early stage and be competitive in the field. However, it can be controlled by applying prochloraz-manganese chloride complex, and this fungicide has no effect on the growth of *Hymenopellis raphanipes*. The results of this study provide essential information for future prevention and control of green mold diseases on *Hymenopellis raphanipes*.

## Data availability statement

The datasets presented in this study can be found in online repositories. The names of the repository/repositories and accession number(s) can be found below: https://www.ncbi.nlm.nih.gov/genbank/, MZ330754-MZ330756, ON074580, ON074583, ON088661-ON088664, ON102005-ON102008, and ON102011.

## Author contributions

X-YZ and F-HT: designing, analyzing, and writing. X-XY, K-QP, Y-TP, T-JT, and NW: collecting specimens and conducting experiments. All authors contributed to the article and approved the submitted version.
