# Supplementary material for: Taxonomy and control of Trichoderma hymenopellicola sp. nov. responsible for the first green mold disease on Hymenopellis raphanipes
Source: Front Microbiol. 2022 Sep 29;13:991987. doi: 10.3389/fmicb.2022.991987 (PMC9559395; doi:10.3389/fmicb.2022.991987)
Supplement: Supplementary file 1 [file Table_1.DOCX]

**Supplementary Table 1**. Sequences used for phylogenetic analyses.

| **Species** | **Strain** | **ITS** | **RPB2** | **TEF** |
| --- | --- | --- | --- | --- |
| *T. achlamydosporum* | YMF 1.06226 | MN977791 | MT052180 | MT070156 |
| *T. aerugineum* | CBS 120541 T | FJ860720 | FJ860516 | FJ860608 |
| *T. afarasin* | DIS 314F | FJ442259 | FJ442778 | FJ463400 |
| *T. afroharzianum* | CBS 466.94 | KP009262 | KP009150 | KP008851 |
| *T. aggregatum* | HMAS 248863 | KY687946 | KY688001 | KY688062 |
| *T. aggressivum* | CBS 100525 |  | AF545541 | AF534614 |
| *T. alni* | CBS 120633 T | EU518651 | EU498349 | EU498312 |
| *T. alpinum* | HMAS 248821 T | KY687906 | KY687958 | KY688012 |
| *T. amazonicum* | IB 95 |  | HM142368 | HM142377 |
| *T. anaharzianum* | YMF 1.00383 | MH113931 | MH158995 | MH183182 |
| *T. asiaticum* | YMF 1.00352 | MH113930 | MH158994 | MH183183 |
| *T. atrobrunneum* | S3 |  | KJ665241 | KJ665376 |
| *T. atrogelatinosum* | CBS 237.63 T | MH858272 | KJ842201 | KJ871083 |
| *T. attinorum* | LESF 236 |  | KT278971 | KT279039 |
| *T. aureoviride* | CPK 2848 | FJ860733 | FJ860523 | FJ860615 |
| *T. azevedoi* | CEN1422 T | MK714902 | MK696821 | MK696660 |
| *T. bannaense* | HMAS 248840 T | KY687923 | KY687979 | KY688037 |
| *T. breve* | HMAS 248844 T | KY687927 | KY687983 | KY688045 |
| *T. brevicrassum* | HMAS 248871 T | KY687954 | KY688008 | KY688064 |
| *T. britannicum* | CBS 253.62 T | MH858149 | KF134787 | KF134796 |
| *T. brunneoviride* | CBS 121130 | EU518659 | EU498357 | EU498316 |
| *T. byssinum* | HMAS 248838 T | KY687921 | KY687977 | KY688035 |
| *T. catoptron* | GJS 02-76 T | AY737766 | AY391900 | AY391963 |
| *T. ceraceum* | GJS 95-159 T | AF275332 | AF545508 | AF534603 |
| *T. ceramicum* | CBS 114576 T | FJ860743 | FJ860531 | FJ860628 |
| *T. ceratophylli* | YMF 1.04621 | MK327581 | MK327580 | MK327579 |
| *T. cerinum* | S357 |  | KF134788 | KF134797 |
| *T. chlamydosporicum* | HMAS 248850 | KY687933 | KY687989 | KY688052 |
| *T. chlorosporum* | GJS 88-33 T |  | AY391903 | AY391966 |
| *T. christiani* | CBS 132572 T |  | KJ665244 | KJ665439 |
| *T. chromospermum* | HMAS 252535 | KF923304 | KF923315 | KF923292 |
| *T. cinnamomeum* | GJS 97-237 | AY737759 | AY391920 | AY391979 |
| *T. compactum* | CBS 121218 T |  | KF134789 | KF134798 |
| *T. concentricum* | HMAS 248833 T | KY687915 | KY687971 | KY688027 |
| *T. corneum* | GJS 97-82 ET |  | KJ665252 | KJ665455 |
| *T. costaricense* | PC 21 T | AY737754 | AY391921 | AY391980 |
| *T. cremeoides* | S112 T |  | KJ665253 | KJ665456 |
| *T. cremeum* | GJS 91-125 T | AY737760 | AF545511 | AF534598 |
| *T. cuneisporum* | GJS 91-93 T | AY737763 | AF545512 | AF534600 |
| *T. dacrymycellum* | WU 29044 | FJ860749 | FJ860533 | FJ860633 |
| *T. danicum* | CBS 121273 T | FJ860750 | FJ860534 | FJ860634 |
| *T. epimyces* | CBS 120534 T | EU518663 | EU498360 | EU498320 |
| *T. estonicum* | GJS 96-129 T | AY737767 | AF545514 | AF534604 |
| *T. ganodermatis* | HMAS 248856 | KY687939 | KY687995 | KY688060 |
| *T. gelatinosum* | GJS 88-17 | AY737775 | AF545516 | AF534579 |
| *T. gliocladium* | CBS 130009 T | MH865622 | KJ665271 | KJ665502 |
| *T. guizhouense* | S278 |  | KF134791 | KF134799 |
| *T. hainanense* | HMAS 248837 T | KY687920 | KY687976 | KY688033 |
| *T. harzianum* | CBS 226.95 T | AY605713 | AF545549 | AF534621 |
| *T. hausknechtii* | CBS 133493 T |  | KJ665276 | KJ665515 |
| *T. helicolixii* | CBS 133499 T |  | KJ665278 | KJ665517 |
| *T. helicum* | DAOM 230021 |  | DQ087239 | KJ871125 |
| *T. hirsutum* | HMAS 248834 T | KY687916 | KY687972 | KY688029 |
| *T. hunanense* | HMAS 248841 T | KY687924 | KY687980 | KY688039 |
| ***T. hymenopellicola*** | **GUCC202008** | **MZ330754** | **ON088663** | **ON102007** |
| ***T. hymenopellicola*** | **GUCC202009** | **MZ330755** | **ON088664** | **ON102008** |
| ***T. hymenopellicola*** | **GUCC202010** | **MZ330756** | **ON088661** | **ON102005** |
| ***T. hymenopellicola*** | **GUCCTB626** | **ON074580** | **ON088662** | **ON102006** |
| ***T. hymenopellicola*** | **GUCCTB625** | **ON074583** | | **ON102011** |
| *T. inaequilaterale* | YMF 1.06203 | MN977795 | MT052186 | MT070152 |
| *T. ingratum* | HMAS 248822 T | KY687917 | KY687973 | KY688018 |
| *T. inhamatum* | CBS 273.78 T |  | FJ442725 | AF348099 |
| *T. italicum* | CBS 132567 T |  | KJ665282 | KJ665525 |
| *T. jaklitschii* | CP61-2 T |  | MW480149 | MW480140 |
| *T. lentiforme* | DIS 94D |  | FJ442749 | FJ463379 |
| *T. lentinulae* | CGMCC 3.19847 T | | MN605867 | MN605878 |
| *T. liberatum* | HMAS 248831 T | KY687913 | KY687969 | KY688025 |
| *T. linzhiense* | HMAS 248846 T | KY687929 | KY687985 | KY688047 |
| *T. lixii* | CBS 110080 T | AF443920 | KJ665290 | FJ716622 |
| *T. longibrachiatum* | CBS 816.68 T | Z31019 | DQ087242 | EU401591 |
| *T. longifialidicum* | LESF 552 |  | KT278955 | KT279020 |
| *T. longipile* | DAOM 177227 T | AY865630 | AF545550 | AF534622 |
| *T. longisporum* | HMAS 248843 | KY687926 | KY687982 | KY688043 |
| *T. lycogaloides* | WU 32096 T |  | KF134792 | KF134800 |
| *T. parepimyces* | CBS 122769 T | FJ860800 | FJ860562 | FJ860664 |
| *T. parestonicum* | CBS 120636 T | FJ860803 | FJ860565 | FJ860667 |
| *T. peberdyi* | CEN1426 T | MK714906 | MK696825 | MK696664 |
| *T. peruvianum* | CP15-2 T |  | MW480153 | MW480145 |
| *T. perviride* | HMAS 273786 T | | KX026962 | KX026954 |
| *T. phyllostachydis* | CBS 114071 T | FJ860809 | FJ860570 | FJ860673 |
| *T. pinicola* | SFC20130926-S233 T | MH050354 | MH025993 | MH025981 |
| *T. pleuroti* | CBS 124387 T |  | HM142372 | HM142382 |
| *T. pleuroticola* | CBS 124383 T |  | HM142371 | HM142381 |
| *T. polypori* | HMAS 248855 T | KY687938 | KY687994 | KY688058 |
| *T. priscilae* | CBS 131487 T |  | KJ665333 | KJ665691 |
| *T. propepolypori* | YMF 1.06224 | MN977789 | MT052181 | MT070158 |
| *T. pseudoasiaticum* | YMF 1.06200 T | MN977792 | MT052183 | MT070155 |
| *T. pseudocandidum* | PC 59 T | AY737757 | AY391899 | AY737742 |
| *T. pseudodensum* | HMAS 248828 T | KY687910 | KY687967 | KY688023 |
| *T. pseudogelatinosum* | CNU N309 T |  | HM920173 | HM920202 |
| *T. purpureum* | HMAS 273787 T | | KX026961 | KX026953 |
| *T. pyramidale* | CBS 135574 T |  | KJ665334 | KJ665699 |
| *T. rifaii* | DIS 337F ET |  | FJ442720 | FJ463321 |
| *T. rosulatum* | HMAS 252548 | KF729995 | KF730005 | KF729984 |
| *T. rufobrunneum* | HMAS 266614 T | KF729998 | KF730010 | KF729989 |
| *T. rugulosum* | SFC20180301-1 T | MH050353 | MH025986 | MH025984 |
| *T. shennongjianum* | HMAS 245009 |  | KT735259 | KT735253 |
| *T. silvae-virgineae* | CBS 120922 |  | FJ860587 | FJ860696 |
| *T. simile* | YMF 1.06201 | MN977793 | MT052184 | MT070154 |
| *T. simmonsii* | S7 |  | KJ665337 | KJ665719 |
| *T. simplex* | HMAS 248842 T | KY687925 | KY687981 | KY688041 |
| *T. sinuosum* | CPK 1595 | FJ860838 | FJ179619 | FJ860697 |
| *T. solum* | HMAS 248848 T | KY687931 | KY687987 | KY688050 |
| *T. spinulosum* | CBS 311.50 T | FJ860844 | FJ860591 | FJ860701 |
| *T. spirale* | DAOM 183974 T | EU280068 | AF545553 | EU280049 |
| *T. stipitatum* | HMAS 266612 | KF730002 | KF730011 | KF729990 |
| *T. stramineum* | GJS 02-84 T | AY737765 | AY391945 | AY391999 |
| *T. strictipile* | CPK 1601 |  | FJ860594 | FJ860704 |
| *T. subazureum* | YMF 1.06207 | MN977799 | MT052190 | MT070148 |
| *T. subuliforme* | YMF 1.06204 | MN977796 | MT052187 | MT070151 |
| *T. sulawesense* | GJS 85-228 |  | AY391954 | AY392002 |
| *T. surrotundum* | GJS 88-73 T | AY737769 | AF545540 | AF534594 |
| *T. tawa* | GJS 97-174 T | AY737756 | AY391956 | AY392004 |
| *T. tenue* | HMAS 273785 T | | KX026960 | KX026952 |
| *T. thailandicum* | GJS 97-61 T | AY737772 | AY391957 | AY392005 |
| *T. thelephoricola* | CBS 120925 | FJ860858 | FJ860600 | FJ860711 |
| *T. tibetense* | HMAS 245010 |  | KT735261 | KT735254 |
| *T. tomentosum* | CBS 120637 |  | FJ860532 | FJ860629 |
| *T. tropicosinense* | HMAS 252546 | KF923302 | KF923313 | KF923286 |
| *T. undatipile* | HMAS 248854 | KY687937 | KY687993 | KY688056 |
| *T. velutinum* | CPK 298 T |  | KF134794 | KJ665769 |
| *T. vermifimicola* | CGMCC 3.19694 T | MN594473 | MN605871 | MN605882 |
| *T. virens* | DAOM 167652 T | EU330955 | AF545547 | AF534619 |
| *T. virescentiflavum* | PC 278 | AY737768 | AY391959 | AY392007 |
| *T. xixiacum* | CGMCC 3.19697 T | MN594476 | MN605874 | MN605885 |
| *T. zayuense* | HMAS 248835 T | KY687918 | KY687974 | KY688031 |
| *T. zelobreve* | CGMCC 3.19695 T | MN594474 | MN605872 | MN605883 |
| *T. zeloharzianum* | YMF 1.00268 | MH113932 | MH158996 | MH183181 |
